# Supplementary material for: Prevalence and diversity of TAL effector-like proteins in fungal endosymbiotic Mycetohabitans spp
Source: Microb Genom. 2024 Jun 11;10(6):001261. doi: 10.1099/mgen.0.001261 (PMC11261895; doi:10.1099/mgen.0.001261)
Supplement: Uncited Supplementary Material 1. [file mgen-10-01261-s001.pdf]

Table S1. *btl* genes and gene fragments (gray background) in ten *Mycetohabitans* spp. genomes.

Fungal Strain	Bacterial Strain	Gene Name[1]	Replicon	Locus	Coordinates	Translated Protein RVDs
ATCC 62417	HKI 0454/ B1	*btl21-1*	Plasmid 1	RBRH_01844	565327...567642	YD NI NI NN NT NN NI NI NN ND NI NI NT NR NS ND NN NG NG NS N*
		btl28-1	Plasmid 2	RBRH_01770	106351...109344	YD NG ** NN NG ** NN NI NS NI NS NI NN NG ** NK NA NI NS NI NI NI ND NS NG KG NT N*
		btl7-1	Plasmid 2	RBRH_01778	104797...105732	HD NG NI NG NG NG NN
ATCC 52813	HKI 0402/ B4/B13	*btl19-13*	Chromosome (CP132744)	RA167_03370	728644...730788	YD ND NI ND NN NI NG NG ** ND NN NN NS NG NG NN NN NI N*
		-	Chromosome (CP132744)	RA167_03350	723396...723821	-
ATCC 52814	HKI 0403/ B7/B14	*btl15-14c*	Chromosome (CP132741)	RA166_09505	2089484..2091232	YD ND NI NI NG ** ND NN NN NS NG NG NN NI N*
		btl15-14p	Plasmid 2 (CP132743)	RA166_16070	95736..97463	YD NG NG NG NG NI NI NI NI NI NI SG NN NN NI
		btl18-14	Plasmid 2 (CP132743)	RA166_16105	106266..108311	YD NG NI NI NG ** ND ND NI NS NN NS NG NG NN NI N*
		-	Chromosome (CP132741)	RA166_09530	2097598..209713	ND NG NN
CBS 111563	HKI 0455/ B3	*btl8-3*	Chromosome (CP062181)	IHE27_12345	2068391..2069461	YD NG NN NG ND NI NI N*
CBS 112285	HKI 0456/ B5	-	Plasmid 1 (CP062179)	IHE28_14160	612407..612753	-
ATCC 52812	B12	*btl28-12*	Plasmid 2 (CP062177)	IHE29_16130	68949..71942	YD NG ** NN NG ** NN NI NS NI NS NI NN NG ** NK NA NI NS NI NI NI NG NS NG KG NT N*
		btl21-12	Plasmid 2 (CP062177)	IHE29_16140	72678..74996	YD NI NI NN NT NN NI NI NN ND NI NI NI NR NS ND NN NG NG NS N*
		btl8-12	Plasmid 2 (CP062177)	IHE29_16125	67296..68330	HD NG NG IG NG NG NG NS
		-	Plasmid 2 (CP062177)	IHE29_16110	63680..63913	-

NRRL 5546	B46	-	Contig 3	IHE30_05195	280204..280683	-
		-	Contig 8	IHE30_10450	136858..137049	-
NRRL 5547	B47	*btl18-47*	Plasmid 1 (CP062173)	IHE31_00180	45803...47848	YD ND NI NI NG ** ND ND NS NS NN NS NG NG NN NN NI N*
		btl21-47	Plasmid 1 (CP062173)	IHE31_02255	598438...600753	YD NI NI NN NT NN NI NI NN ND NI NI NI NR NS ND NN NG NG NT N*
		btl13-47	Plasmid 1 (CP062173)	IHE31_00165	40437...41966	YD NG NN NG ND NI NI NI NI SG NN NN NI
		-	Chromosome (CP062174)	No ORF	638061..641324	-
NRRL 5549	B49	*btl28-49*	Chromosome (CP062171)	IHE32_09390	2124213..2127206	YD NG ** NN NG ** NN NI NS NI NS NI NN NG ** NK NA NI NS NI NI NI NG NS NG KG NT N*
		btl21-49	Plasmid 1 (CP062172)	IHE32_14660	621296..623611	YD NI NI NN NT NN NI NI NN ND NI NI NT NR NS ND NN NG NG NS N*
		-	Chromosome (CP062171)	No ORF	590914..591150	-
NRRL 5560	B60	*btl20-60*	Plasmid 1 (CP062169)	IHE33_14225	506369...508612	YD NG ND NI ND NN NI NG NG ** ND NN NI NI NG NG NN NN NV N*
		btl14-60	Plasmid 1 (CP062169)	IHE33_14240	512461...514089	YD NG NG NG NG NI NI NI NI SG NN NN NI NN
		-	Chromosome (CP062168)	IHE33_09065	2121146..2121352	-
		btl8-60[2]	Chromosome (CP062168)	IHE33_09045	2117750..2118772	NI NS NG NG NN NN NV N*
		-	Plasmid 2 (CP062170)	IHE33_15585	102460..103052	-

[1]*btl* gene names are the repeat number, a dash, then the strain number. Repeat number includes the conserved YD/HD repeat that precedes specificity-defining repeats. The letters p and c distinguish plasmid- vs. chromosomally-located genes in a strain if the genes have the same number of repeats.

[2]Interrupted by a transposase gene insertion at the 5’ end.

Table S2. Predicted type III secretion signal and putative *hrp_{II}* box for full-length *btl* genes from the seven newly sequenced *Mycetohabitans* genomes.

**btl *genes***	***Locus***	***Predicted T3SS signal[a]***	***hrp_{II} Box Sequence (TTCG-N16-TTCG)***	***hrp_{II} box position***	***Distance between hrp_{II} box and ATG***
btl8-3	IHE27_12345	None	CGAACGCTTCACCTACTGGGCGAA	2069677..2069700	215
btl28-12	IHE29_16130	0.99902	TTCGCTCAGTAGGTGAAGCGTTCG	68710..68733	215
btl21-12	IHE29_16140	None	TTCGCCCAGTAGGTGAAGCGTTCG	72449..72472	205
btl8-12	IHE29_16125	None	TTCGCCGGATCGGCGGTGCGTTCG	67076..67099	196
btl21-8	IHE29_16125	None	TTCGCCGGATCGGCGGTGCGTTCG	67076..67099	196
btl18-5547	IHE31_00180	None	TTCGCCCAGTAGGTGAAGCGTTCG	45564...45587	215
btl21-5547	IHE31_02255	None	TTCGTTCAGCACGCAATGCGTTCG	600960..600983	206
btl13-5547	IHE31_00165	None	TTCGCCGGATCGGCGGCGCGTTCG	40217...40240	196
btl28-5549	IHE32_09390	0.99902	TTCGCCCAGTAGGTGAAGCGTTCG	2127422...2127445	215
btl21-5549	IHE32_14660	None	TTCGTTCAGCACGCAATGCGTTCG	623818..623841	205
btl20-5560	IHE33_14225	0.99902	TTCGCCCAGTAGGTGAAGCGTTCG	508828..508851	215
btl14-5560	IHE33_14240	None	TTCGCCGGATCGGCGGCGCGTTCG	514286..514309	196

[a] T3SS signal prediction was carried out using EffectiveDB (36).

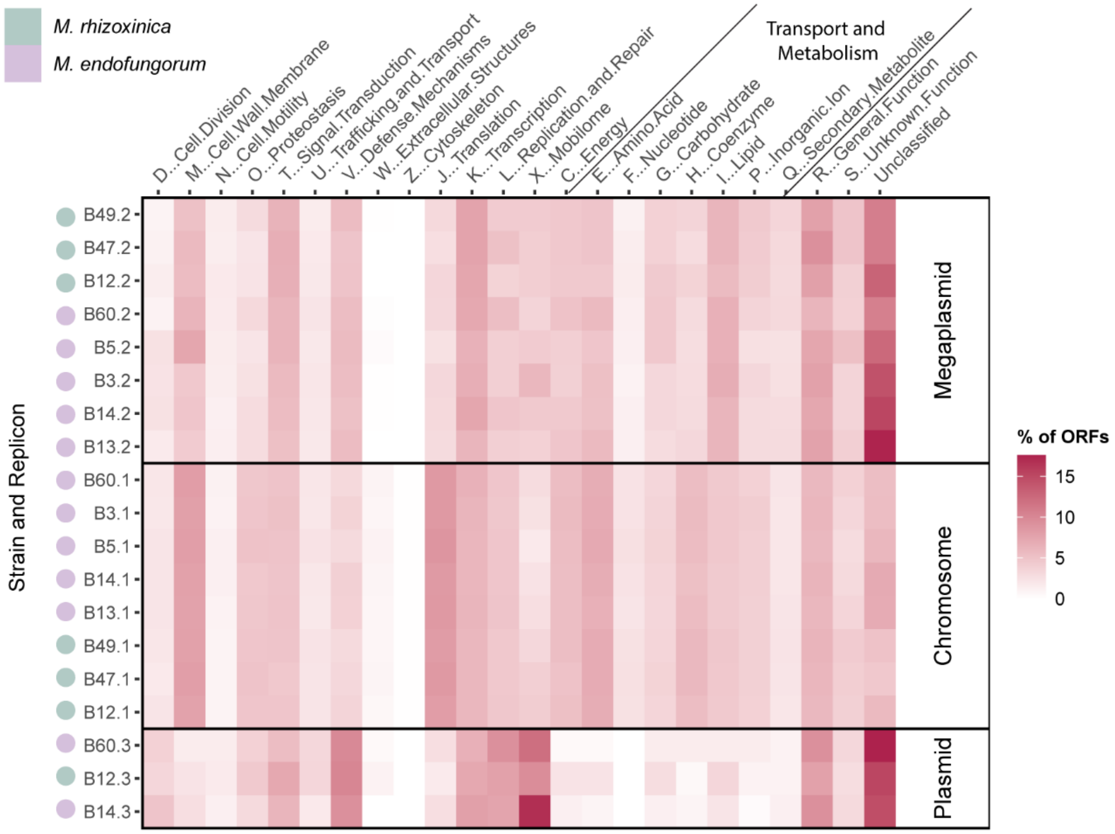

Figure S1. Clusters of Orthologous Genes (COGs) analysis of the chromosomes and megaplasmids of completely assembled genomes showing the percent of open reading frames (% of ORFs) from each replicon assigned to each COG. Replicons are indicated by strain name and number, with a colored dot to the left indicating the species of that strain.

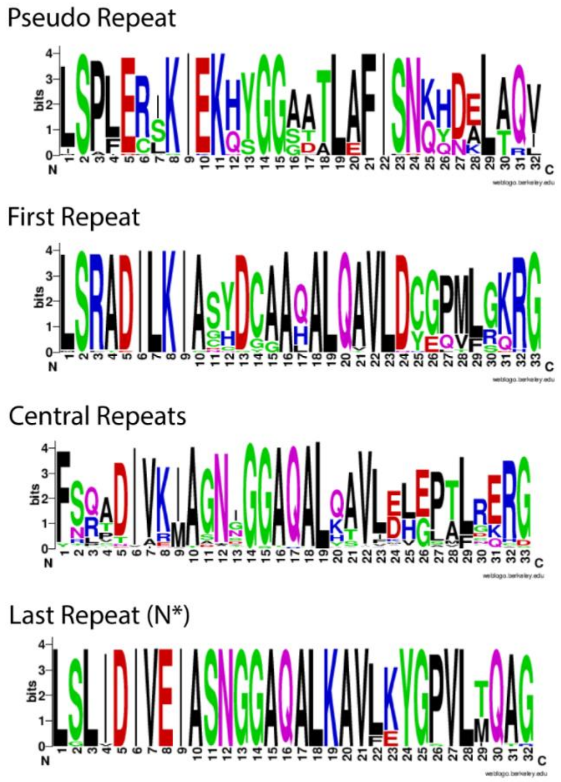

Figure S2. Sequence logo motifs for the pseudo repeats, first repeats, central repeats, and terminal repeats of all intact Btl proteins used in Figure 3. For the last repeats, only those with an N* RVD (the asterisk denoting a missing residue) were included, as those with other RVDs are indistinguishable from central repeats.

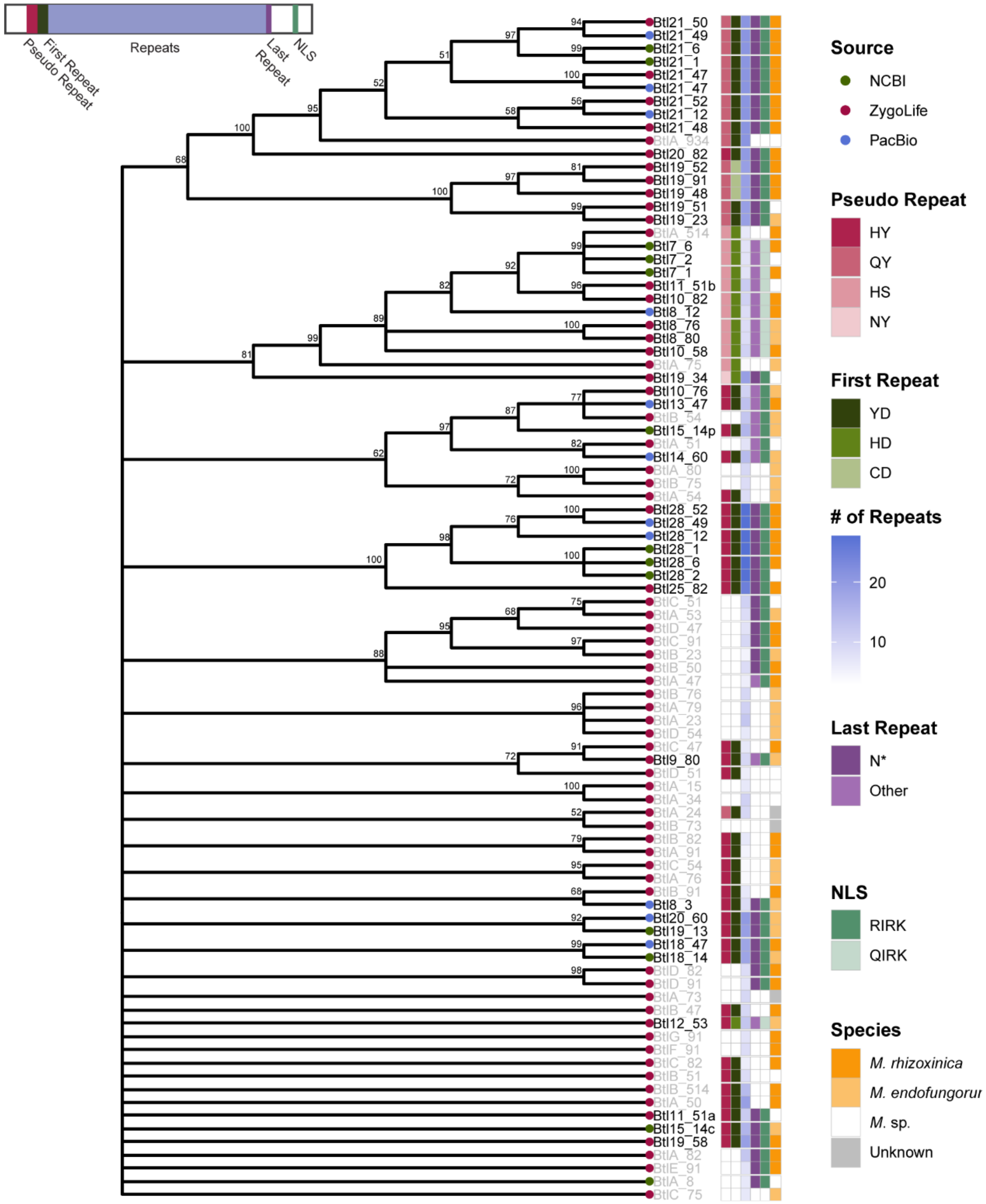

Figure S3. Maximum likelihood phylogenetic analysis of encoded Btl protein sequences across 32 isolates. Grey tip labels indicate incomplete protein sequences, typically found encoded at the end of an assembled contig; these are named using a letter in place of the repeat number. Branches with bootstrap values less than 50% were collapsed; those with >50% are labeled at the nodes. As shown in the keys on the right, colored circles at tips indicate the source of the data for each sequence, and colored boxes detail *Mycetohabitans* species, number of repeats, and presence or absence of key protein motifs, including the nuclear localization signal (NLS), shown using the single letter amino acid code. A diagram of a Btl protein with motif locations is in the top left.
